# Settling Decisions and Heterospecific Social Information Use in Shrikes

**DOI:** 10.1371/journal.pone.0003930

**Published:** 2008-12-11

**Authors:** Martin Hromada, Marcin Antczak, Thomas J. Valone, Piotr Tryjanowski

**Affiliations:** 1 Department of Zoology, Faculty of Science, University of South Bohemia, České Budějovice, Czech Republic; 2 Department of Behavioural Ecology, Adam Mickiewicz University, Poznań, Poland; 3 Department of Biology, Saint Louis University, St. Louis, Missouri, United States of America; University of Utah, United States of America

## Abstract

Animals often settle near competitors, a behavior known as social attraction, which belies standard habitat selection theory. Two hypotheses account for these observations: individuals obtain Allee benefits mediated by the physical presence of a competitor, or they use successfully settled individual as a source of information indicating the location of high quality habitat. We evaluated these hypotheses experimentally in two species of shrikes. These passerine birds with a raptor-like mode of life impale prey to create larders that serve as an indicator of male/habitat quality. Thus, two forms of indirect information are available in our system: a successfully settled shrike and its larder. Typically these two cues are associated with each other, however, our experimental treatment created an unnatural situation by disassociating them. We manipulated the presence of larders of great grey shrikes and examined the settling decisions of red-backed shrikes within and outside the great grey shrike territories. Male red-backed shrikes did not settle sooner on plots with great grey shrikes compared to plots that only contained artificial larders indicating that red-backed shrikes do not use the physical presence of a great grey shrike when making settling decisions which is inconsistent with the Allee effect hypothesis. In contrast, for all plots without great grey shrikes, red-backed shrikes settled, paired and laid clutches sooner on plots with larders compared to plots without larders. We conclude that red-backed shrikes use larders of great grey shrikes as a cue to rapidly assess habitat quality.

## Introduction

According to habitat selection theory the fitness of animals in a habitat patch is assumed to be negatively density-dependent, i.e., fitness is reduced by the presence of competitors [1]. In numerous taxa, however, individuals often exhibit social attraction: a preference of settling near competitors that may either be conspecifics or heterospecifics - phenomena known as conspecific and heterospecific attraction, respectively [2]–[11].

Two general hypotheses explain why individuals might benefit by settling near competitors. The first posits that individuals obtain direct fitness benefits such as reduced predation risk, better defense against intruders, increased feeding benefits and/or social stimuli which improves their mating opportunities [2], [4], [7], [10], [12], [13]. Because fitness is positively density dependent (higher fitness for individuals that settle near others), these benefits are examples of an Allee effect [2], [4], [14]. A central aspect of this hypothesis is that such Allee effect benefits are mediated by the physical presence of other individuals.

The second hypothesis suggests that animals use the presence of a successfully established individual as an indicator of the location of high quality habitat [2], [10], [15]. Individuals that must rapidly assess habitat quality can do so quickly by using the presence of a successfully settled individual as a form of public information [13], [16]–[18]. Because both Allee benefits and information about habitat quality are provided by the presence of a competitor it has been difficult to tease apart these explanations empirically [11], [13], [19].

We examined heterospecific attraction in shrikes to test these hypotheses. Shrikes, small raptor-like passerine birds, create temporary caches by impaling prey on thorns or in forked branches throughout their territory because, unlike raptors, they do not have talons and strong feet, so impaling and wedging is a necessary adaptation for dismembering and handling large prey items. Scattered caches are distributed over a shrike's territory, most frequently in distinctive places, such as at the tops of trees or shrubs on dry or broken sprigs, but also on artificial objects, such as on barbed wire fences [20], [21]. Besides their obvious function as a temporary food cache, larders also can provide information to conspecifics. During territory establishment, larders serve as landmarks of territory boundaries for other conspecific males [22], [23]. In addition, caches also can advertise the quality of a male and/or its territory quality to females. Male shrikes with larger larders are preferred by females [24], [25]. Here, we investigated whether larders might also provide information to heterospecifics.

### System and hypotheses

Red-backed shrikes (*Lanius collurio*) and great grey shrikes (*Lanius excubitor*) share similar breeding habitats: a mix of farmland and natural meadows with dispersed shrubs and trees for nests, perches and cache sites, and often nest near one another [21], [26]–[30]. Both species use similar hunting methods and exploit similar food resources [29], [30], [31]. Therefore, we assume that high quality habitat in terms of prey availability for one species is high quality habitat for the other.

In western Poland, great grey shrikes are year-round territorial residents and one of the earliest breeding passerines [32], [33]. They have large territories (up to 200 ha) that are widely dispersed (on average, 1.8 km separates adjacent territories [32]). In contrast, red-backed shrikes are long-distance migrants, wintering exclusively in Africa [29]. Territories of this species are much smaller (0.4 ha on average) and more closely spaced (on average, 125 m separates adjacent territories [30]).

In Eastern Europe, red-backed shrikes are one of the last arriving species in spring [34] and thus face a shortened breeding season. Males arrive on the breeding grounds prior to females [35], [36] and choose the nesting site. At this time of the year, typically only great grey shrike territories are decorated with larders – all territories of the species contain larders (for more information see [23]); red-backed shrikes occasionally also impale their prey later during breeding season [30]. We hypothesized that red-backed shrikes might preferentially settle near great grey shrike territories (i.e., exhibit heterospecific social attraction) to obtain Allee effect benefits via the presence of a great grey shrike or use the larders of great grey shrikes as a cue to the location of high quality habitat. We manipulated the environment to tease apart these two alternatives. Four types of experimental plots were established: plots with and without artificially added larders both within and outside existing great grey shrike territories. We predicted that if red-backed shrikes seek Allee effect benefits, they should preferentially settle in plots containing great grey shrikes compared to those without. Alternatively, if red-backed shrikes use larders as a cue to habitat quality in terms of prey availability, they should preferentially settle on plots with experimentally added larders compared to plots without, independent of the presence of a great grey shrike.

## Results

For detail information on sample sizes on particular experimental plots see Table 1.

**Table 1:** Numbers of red-backed shrike pairs included into analyses for particular treatment plots (not all study plots were occupied by red-backed shrikes and there were also some minor losses during the breeding season).

	+L−G	C−G	+L+G	C+G	Total
Male arrival date	10	7	11	8	36
Pair formation date	10	7	11	8	36
Laying date	8	7	8	8	31
Total number of pairs on plots	25	10	37	14	86
Plots occupied by red-backed shrikes	10	7	10	8	35

Treatment symbols are as follows: +L: larders added; C: control, no larders added; −G: outside of great grey shrike territory; +G: within great grey shrike territory.

**Figure 1:**
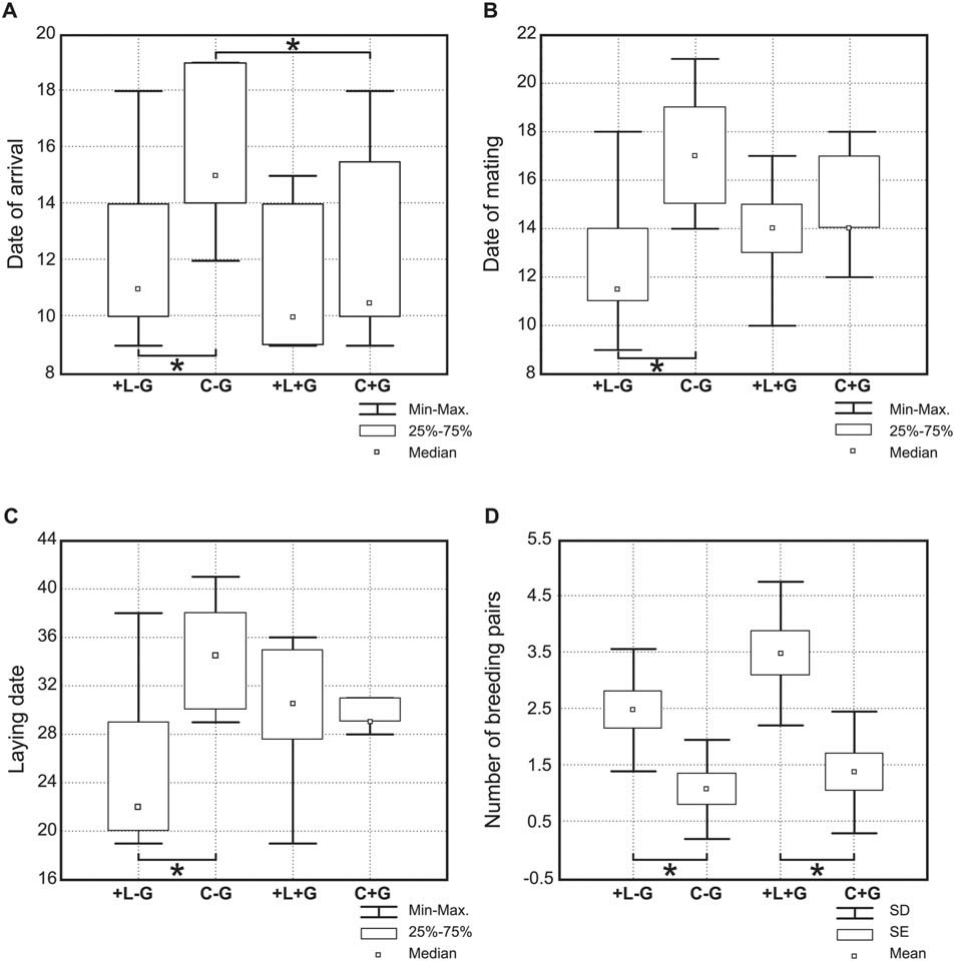
Effect of treatment plots on median a) settling date of male red-backed shrikes (days after May 1^st^ ), b) pairing date (days after May 1^st^ ), c) egg laying date (days after May 1^st^ ) and d) number of red-backed shrike breeding pairs. Significant relationships are marked with asterisks.

Arrival dates of male red-backed shrikes differed significantly among treatment plots (Kruskal-Wallis ANOVA, *H_3,36_* = 8.88, *p* = 0.03, Figure 1A). They settled approximately 5 days earlier on unmanipulated plots with great grey shrikes (C+G) compared to unmanipulated plots without great grey shrikes (C−G), i.e. they exhibited heterospecific attraction (Newman-Keuls test, *p* = 0.048, Figure 1A).

Post-hoc pairwise comparisons of treatment plots allow tests of our predictions. For all plots with larders present (+L−G, +L+G, C+G), red-backed shrikes did not settle earlier on plots with great grey shrikes compared to plots without great grey shrikes (+L+G and C+G vs. +L−G) (Newman-Keuls test, *p* = 0.69 and 0.12 respectively, Figure 1A) which is not consistent with the Allee effect hypothesis prediction. Comparison of the treatment plots without great grey shrikes (+L−G vs. C−G) shows that red-backed shrike males settled significantly earlier on plots that contained an artificial larder (+L−G) (Newman-Keuls test, *p* = 0.02, Figure 1A) as predicted by the information hypothesis.

Female red-backed shrikes also exhibited differences in how quickly they formed pair-bonds with males on treatment plots (Kruskal-Wallis ANOVA, *H_3,36_* = 11.74, *p* = 0.008, Figure 1B). For plots without great grey shrikes, females paired with males on plots with larders (+L−G) 6 days earlier than males on plots without larders (C−G) (Neuman-Keuls test, *p* = 0.05, Figure 1B). However, for plots with great grey shrikes, females did not pair earlier with males on plots with supplemented larders (+L+G) (Neuman-Keuls test, *p* = 0.58, Figure 1B). No unmated red-backed shrikes were observed on study plots.

The differences in pairing date translated into earlier egg laying dates (Kruskal-Wallis ANOVA, *H_3,31_* = 8.72, *p* = 0.03, Figue 1C). Females on plots with artificial larders but no great grey shrike (+L−G) laid clutches up to 12 days sooner than females on plots with no shrike and no larder (C−G) (Neuman-Keuls test, *p* = 0.024, Figure 1C ).

Experimental treatments also affected the total number of red-backed shrikes that settled on the plots (ANOVA, *F_3,35_* = 10.23, *p*<0.001, *n* = 40, Figure 1D). In the absence of great grey shrikes, more red-backed shrikes settled on plots with artificial larders (+L−G) compared to plots without (C−G) (Tukey test, *p* = 0.03) and for plots with great grey shrikes, more red-backed shrikes settled on plots with supplemented larders (+L+G) compared to those without (C+G) (Tukey test, *p*<0.001).

## Discussion

Individuals can use two general kinds of information to assess habitat quality: direct personal information which may involve prior breeding experiences or sampling of habitat quality and indirect information such as the presence of other individuals [4], [22].

In our study region the mortality of red-backed shrikes is high, only 20% of birds survive two years of life [37], therefore they typically breed only once. Moreover, natal and breeding-site fidelity is practically nil [30]. So, males cannot rely on prior information about breeding grounds and breeding success in previous year to assess habitat quality. Our data suggest that male red-backed shrikes apparently rely on indirect information to assess habitat quality because they settled preferentially on plots with great grey shrikes or larders. Previous studies have shown that the larders of great grey shrikes represent signals – evolved advertent social information [38], which serve for intraspecific communication [22]–[25]. However, we found that great grey shrike larders also serve as a source of indirect social information [39] for a different species: red-backed shrikes use them as a habitat cue to select a breeding site. The possibility that red-backed shrikes seek larders only to feed on them seems unlikely because we have observed only three such cases during the experiment.

Our experimental treatments created an unnatural situation by disassociating the presence of a great grey shrike from the presence of a larder to provide insight into how red-backed shrikes make settling decisions. Our data suggest that male red-backed shrikes do not simply use the presence of a great grey shrike to settle on territories because plots with only larders were settled at the same time as plots with larders and great grey shrikes. Thus, the settling decisions of males appear not to be driven by the physical presence of an individual and the Allee effect benefits that would provide. Instead, red-backed shrikes apparently use the presence of great grey shrike larders to assess habitat quality and thus exhibit heterospecific attraction. We suggest that the benefit of doing so is that it allows more rapid habitat assessment and territory establishment which can have strong effects on fitness [40].

Because we did not measure the performance of birds on treatment plots, we cannot rule out the possibility that red-backed shrikes obtain fitness benefits by settling near great grey shrikes.

However, some studies suggests that the inadvertent cues about good quality habitat provided by earlier established species may be the main mechanism behind heterospecific attraction while social interactions with other species are less important [41], [42]. Moreover, the pattern of red-backed shrike density on treatment plots we observed also may indicate that larders serve as a cue to habitat quality: more birds settled in plots with larger larders, both within and outside great grey shrike territories. While we did not quantify precisely the number of larders in great grey shrike territories because of the logistical difficulties involved, the number of larders within great grey shrike territories with added artificial larders was up to twice the typical amount. We suggest that the amount of larders on the plot may, to some extent, serve as an indicator of great grey shrike performance for red-backed shrikes.

We have shown that heterospecific attraction in this system does not require the physical presence of an individual as occurs in other examples of conspecific and heterospecific attraction (e.g., [2]–[11]).

The numerous observations of both conspecific and heterospecific attraction suggest that interactions between competitors are not always negative. In circumstances in which environmental parameters need to be estimated, the presence or behavior of competitors can be a form of information that allows individuals to make more rapid decisions. Similar benefits of public information use have been observed in the contexts of foraging, fighting and nesting [43]–[47]. Our work thus adds to the growing literature demonstrating that competitors can obtain information about environmental quality from the performance and behavior of heterospecifics (e.g., [47]–[49]).

Our findings also suggest that ongoing declines in many populations may have unforeseen negative consequences. Population declines of one species within a community are often assumed to benefit a putative competitor. However, if competitors use one another as a source of information about habitat quality, declines in the abundance of one species may lead to reduced fitness for the other. The growing evidence of heterospecific attraction suggests an additional mechanism to explain concomitant population declines in species within competitive communities [50].

## Materials and Methods

### Experimental procedures

Fieldwork was carried out in 2003 season near Odolanów (51°34′N, 17°40′E), Poland, in an agricultural landscape containing arable fields, meadows, small woodlots and tree rows [28], [32], [33]. In the region studied, both species are common. Both shrike species have been monitored in the area since 1999, mean breeding density of the great grey shrike here reaches 13–15 pairs per 100 km^2^ and red-backed shrike density varies between 5 to 12 breeding pairs per 1 km^2^ [28], [32], [33].

During early spring, the study area was searched for great grey shrike territories and all nests were mapped. In April, we randomly established 40 300×300 m experimental plots in locations where red-backed shrikes as well as great grey shrikes had established territories in previous years. All plots were separated by at least 500 m. Twenty plots contained a great grey shrike territory and 20 did not. To minimize potential pseudoreplication, only one experimental plot was established in each great grey shrike territory. The great grey shrikes from the studied population have a high reoccupation rate of breeding territories, up to 70% of territories are occupied in the following seasons [33]. We added artificial larders to half the plots containing great grey shrikes and half the plots without great grey shrikes. Thus, we had four treatments, each with 10 replicates. The manipulation was performed 3–5 days before arrival of red-backed shrikes. Artificial larders consisted of 20 food items per plot: 14 vertebrate (3–4 cm long slices of commercially sold chicken stomachs, or in several cases, small birds, mammals and frogs found killed on roads) and 6 invertebrate (crickets and large Silphids) prey items, simulating the natural ratio [23]. Since great grey shrikes scatter impaled food caches mostly individually over territory, we dispersed larders throughout the plot similar to the way natural larders are dispersed, in distinctive places, such as on sharp twigs and thorns or in forked branches [23]. After the five week experiment, 60% of impaled items were still present on territories. There are 10–40 larders in typical great grey shrike territory, each consists of one or two impaled prey items [23], so that addition of 20 artificial caches in every manipulated territory created a typical cache in territories without great grey shrikes and increased cache size by approximately 100% in territories with great grey shrikes. We did not observed any desertion of the great grey shrikes in manipulated territories, so we believe that the artificial larders have no effect on territorial behavior of the great grey shrikes.

Beginning May 1^st^, all plots were intensively monitored each day for the arrival of red-backed shrikes, as well as the initiation of mating and nesting behaviour, over the next five weeks. Over the past 20 years, May 1^st^ was the earliest recorded arrival date for red-backed shrikes [34]. We determined settling date by the observation of territorial behaviors such as singing and flight displays. Pairing date was determined by observing one of the following: a male and female sitting close together in a tree or shrub, mating behavior or feeding displays. Egg laying date was estimated either directly by visual inspection of nests or indirectly following the first observation of nestlings and knowledge of the 14 day incubation period that begins after the entire clutch of 4 eggs have been laid and the fact that females lay one egg per day.

All statistical tests were two-tailed, except where indicated. We used Kruskal-Wallis ANOVA with Newman-Keuls post-hoc tests to examine treatment effects on dates of settling, pair bond formation and egg laying.
