# Correction: Settling Decisions and Heterospecific Social Information Use in Shrikes

**DOI:** 10.1371/annotation/872bc5d4-93ed-4cb3-b6ed-539ead778ad8

**Published:** 2008-12-18

**Authors:** Martin Hromada, Marcin Antczak, Thomas J. Valone, Piotr Tryjanowski

The grant number from the funder University of South Bohemia Ceske Budejovice was listed incorrectly. The correct grant number is: MSM6007665801.

